# Supplementary material for: Detailed molecular and epigenetic characterization of the pig IPEC-J2 and chicken SL-29 cell lines
Source: iScience. 2023 Feb 20;26(3):106252. doi: 10.1016/j.isci.2023.106252 (PMC10018572; doi:10.1016/j.isci.2023.106252)
Supplement: Data S2. Complete homer output for identified motifs in Chicken SL-29, related to Tables 5 and 6 — Homer motif analysis results for histone modifications H3K4me1, H3K4me3, H3K27ac, enhancers, and ATAC-seq of chicken SL-29 cell line. Parameters for possible false positives is as mentioned earlier for S5. [file mmc3.zip › Data_S2/S6/Chicken_SL_29/peak_files_H3K4me3/homerResults/motif4.similar.html]

motif4

## Information for motif4

C
T
A
G
G
T
A
C
T
G
A
C
C
A
T
G
G
T
A
C
T
G
C
A
G
T
C
A
G
C
A
T
  
Reverse Opposite:  

C
G
T
A
C
A
G
T
A
C
G
T
C
A
T
G
G
T
A
C
A
C
T
G
C
A
T
G
G
A
T
C
  

|  |  |
| --- | --- |
| p-value: | 1e-87 |
| log p-value: | -2.008e+02 |
| Information Content per bp: | 1.598 |
| Number of Target Sequences with motif | 8467.0 |
| Percentage of Target Sequences with motif | 49.32% |
| Number of Background Sequences with motif | 13631.0 |
| Percentage of Background Sequences with motif | 41.80% |
| Average Position of motif in Targets | 150.6 +/- 87.7bp |
| Average Position of motif in Background | 150.1 +/- 95.8bp |
| Strand Bias (log2 ratio + to - strand density) | -0.0 |
| Multiplicity (# of sites on avg that occur together) | 1.40 |
| Motif File: | file (matrix) reverse opposite |

### Similar de novo motifs found

|  |  |  |  |  |  |  |  |
| --- | --- | --- | --- | --- | --- | --- | --- |
| Rank | Match Score | Redundant Motif | P-value | log P-value | % of Targets | % of Background | Motif file |
| 1 | 0.645 | A T G C A C T G A T G C T A G C C A T G T A G C A T G C C G T A A C G T A T C G A C T G G A T C A T C G | 1e-60 | -139.424652 | 11.35% | 7.77% | motif file (matrix) |
| 2 | 0.693 | A C G T G C T A T G A C G C T A C T A G T A C G G T A C A T G C A C T G G T A C | 1e-60 | -139.136028 | 19.23% | 14.61% | motif file (matrix) |
| 3 | 0.665 | A C T G G T A C T A G C G C T A C A G T C T A G A C T G G A T C A T C G T A C G | 1e-58 | -134.366711 | 14.99% | 10.95% | motif file (matrix) |
| 4 | 0.630 | G T C A C A T G G T A C T C A G A T C G G T A C G T C A G C A T | 1e-57 | -132.926155 | 15.12% | 11.08% | motif file (matrix) |
| 5 | 0.694 | T C G A G C A T A T G C T A G C A T C G T G C A T C G A C T G A | 1e-49 | -114.183901 | 48.24% | 42.60% | motif file (matrix) |
| 6 | 0.612 | G A T C C G T A A C T G T G A C G T C A C T A G T A G C G T A C | 1e-38 | -89.174369 | 51.43% | 46.44% | motif file (matrix) |
| 7 | 0.653 | G T A C A T C G A C T G T A G C A G C T C G T A G A C T G C T A | 1e-38 | -88.922517 | 19.86% | 16.09% | motif file (matrix) |
